# Progress in Microneedle-Mediated Protein Delivery

**DOI:** 10.3390/jcm9020542

**Published:** 2020-02-17

**Authors:** Rezvan Jamaledin, Concetta Di Natale, Valentina Onesto, Zahra Baghban Taraghdari, Ehsan Nazarzadeh Zare, Pooyan Makvandi, Raffaele Vecchione, Paolo Antonio Netti

**Affiliations:** 1 Center for Advanced Biomaterials for Health Care, Istituto Italiano di Tecnologia (IIT@CRIB), 80125 Naples, Italy; Rezvan.jamaledin@iit.it (R.J.); Valentina.onesto@iit.it (V.O.);; 2 Department of Chemical, Materials and Industrial Production Engineering, University of Naples Federico II, 80125 Naples, Italy; zahrabaghban7@gmail.com; 3 School of Chemistry, Damghan University, P.O. Box: 36716-41167, Damghan, Iran; ehsan.nazarzadehzare@gmail.com; 4 Institute for polymers, Composites and biomaterials (IPCB), National research council (CNR), 80125 Naples, Italy; 5 Chemistry Department, Faculty of Science, Shahid Chamran University of Ahvaz, P.O. Box: 61537-53843, Ahvaz, Iran

**Keywords:** microneedles, transdermal, drug delivery, protein, antigen delivery

## Abstract

The growing demand for patient-compliance therapies in recent years has led to the development of transdermal drug delivery, which possesses several advantages compared with conventional methods. Delivering protein through the skin by transdermal patches is extremely difficult due to the presence of the stratum corneum which restricts the application to lipophilic drugs with relatively low molecular weight. To overcome these limitations, microneedle (MN) patches, consisting of micro/miniature-sized needles, are a promising tool to perforate the stratum corneum and to release drugs and proteins into the dermis following a non-invasive route. This review investigates the fabrication methods, protein delivery, and translational considerations for the industrial scaling-up of polymeric MNs for dermal protein delivery.

## 1. Introduction

Proteins, as drugs, play key roles in the treatment of several diseases, such as inflammation or neurodegenerative disorders, cancer, vaccinations, genetic diseases, and so on. They possess several functions in the body, e.g., as enzymes, immunostimulators, cellular regulators, or molecular transporters [1]. Currently, the main route of protein administration is parenteral intake, but this is associated with poor patient compliance and, often, with protein instability [2], even though several approaches have been studied to increase proteins’ in vitro/in vivo stability, such as chemical modifications (PEGylation, hyperglycosylation, mannosylation) or colloidal delivery systems including micro/nanoparticles, liposomes, carbon nanotubes, or the new generation protein-based thermosensitive gels [3]. Other drawbacks exist with regard to oral delivery, where proteins are also subject to gastrointestinal degradation or low bioavailability, limiting their therapeutic efficiency [4]. In this framework, the skin seems to be an effective alternative, although the presence of the stratum corneum limits the delivery of large molecular weight proteins [5]. To pass this biological barrier, several methods have been proposed, such as laser, thermal, or radiofrequency ablation or electrically assisted enhancement techniques such as electroporation and iontophoresis [6]. Unfortunately, all of these techniques seriously damage the skin, inflicting pain on the patients. 

To overcome these limitations, micrometric length microneedles (MNs) have emerged. They are capable of penetrating the epidermis layer at a fixed depth, avoiding the stimulation of the nerve endings; in this way, the macromolecules can be rapidly absorbed in the capillaries and lymphatic networks without producing pain for the patients [7]. They also enable the direct delivery of vaccines into the skin, where dermal dendritic cells (DDCs) are densely distributed, acting as a potent physical adjuvant for successful transcutaneous immunization (TCI) [8]. Moreover, the use of microneedles likewise brings many benefits in cancer treatment, such as easy controllability and applicability, and a major synergistic effect [9]. Other important applications have been developed in the cosmetic field, where treatment with microneedles has made great progress. In particular, microneedles create transient holes to enhance the penetration of cosmetic compounds, and they trigger the wound repair mechanism [9]. 

In addition, MNs are able to encapsulate proteins with high efficiency and store them in a bioactive state without requesting further expedients (cold-chain), hence minimizing the cost of transportation [10]. Moreover, the addition of excipients, such as ethylene-diaminetetraacetic acid trehalose or mannitol, into their formulation can also stabilize the activity of the drugs over several days [11]. Unfortunately, the current MN preparations still show some limitations. For example, solid MNs have expensive fabrication costs and their difficult application processes are troublesome for patients [12]. Coated MNs are able to load only a small amount of drugs since they can only be applied on the surface of the MNs, while hollow MNs are characterized by a potential toxic effect because of the uncontrolled drug dose release that occurs [13,14], and they require specialized personnel and a complex set up for the injection. In addition, there are dissolving MNs, which are not able to provide prolonged release, and most of the methods are not suitable for protein stability [15].

Despite these restrictions, the progress in MN fabrication and protein encapsulation make microneedles a promising platform for the dermal delivery of drugs. In the following chapters, fabrication methods, protein delivery, and translational considerations for the industrial scaling-up of polymeric MNs are discussed.

## 2. Fabrication Methods

### 2.1. Mold-Based Methods

Micromolding is the most commonly used methodology for the fabrication of microneedles. It involves the fabrication of a master with the desired microstructures from which molds can be obtained. Then, after mold fabrication, microneedles can be replicated on it. MN masters are usually produced by techniques such as photolithography using deep X-ray lithography of Lithographie Galvanoformung Abformung [13,16] and ultraviolet (UV) lithography [17], laser ablation [18], micromilling and microgrinding [19,20], additive manufacturing [21,22], laser percussion drilling [13,16], and deep reactive ion etching (DRIE) [23]. The produced master can be re-used to make multiple molds, and each mold can be used several times for MN fabrication after appropriate cleaning.

**Figure 1:**
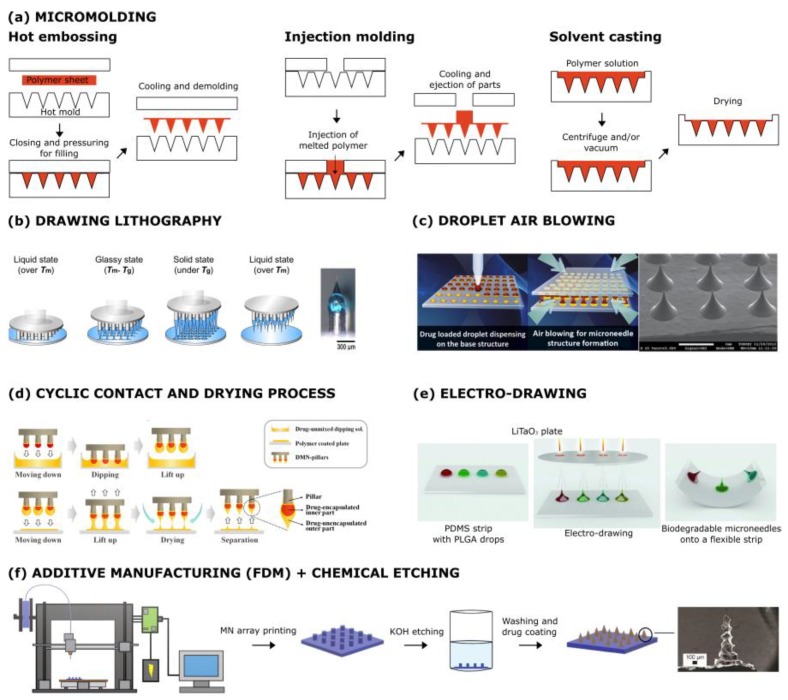
Microneedle (MN) manufacturing methods. (**a**) Micromolding: The mold with the desired MN structures can be filled with polymers by hot embossing, injection molding, or solvent casting. (**b**) Drawing lithography: The polymer is melted, dispensed on a fixed plate, and elongated by pillars in the upper-moving plate. (**c**) Droplet air blowing: Two plates, with polymer drops within, are contacted and then moved. When the final distance between the plates is reached, the polymer is hardened by means of air blowing. (**d**) Cyclic contact and drying: Pillars are repeatedly contacted with a drug-polymer solution, lifted, and dried with air blowing. (**e**) Electro-drawing: A thermal stimulus is applied to a pyroelectric crystal, generating an electric field which drives the microneedle drawing process. (**f**) Fused deposition modeling (FDM) of biodegradable polymer MNs: FDM is followed by KOH etching to improve feature size. Reprinted with modification from [36,37,38,39,40,41].

Micromolding includes hot embossing, injection, and solvent casting (Figure 1a). In hot embossing, the mold needs to be firstly heated to a temperature above the glass transition temperature (Tg) of the polymer, allowing the pressed polymer flow to fill the mold microcavities. The mold is then cooled to a temperature lower than the Tg [23,24,25]. Microinjection molding involves the injection of the melted polymer, forcing it into the mold and subsequently cooling and solidifying the polymer. This is pursued by using an injection molding machine which is divided into two units: a clamping unit and an injection unit. Injection molding results depend on process parameters such as temperature, injection speed, pressure, clamping force, and decompression velocity [26,27,28]. The involvement of high temperatures makes this process unsuitable for the direct encapsulation of protein, limiting the application to post-encapsulation. In solvent casting, a polymer solution is added into the mold, and after that, the vacuum and/or centrifugation steps are performed to fill the mold microcavities [18,19,21,29,30,31,32]. Long evaporation times may compromise the activity of the protein, in addition to being economically expensive [33]. Alternatively, spray coating can be used to deposit polymer solutions into MN molds [20,34] in a faster and safer way for the protein. Finally, the solution in the mold is left to become dry to ensure complete evaporation of the solvent. The drying step can be replaced by photopolymerization if a photocrosslinkable material is used [35] to speed up the process and make it less time consuming and, therefore, less expensive. On the other hand, the UV treatment can inhibit the activity of the active compound encapsulated within the polymer matrix as well as the photoinitiator residuals within the final microneedles, which can introduce danger in terms of toxicity.

### 2.2. Mold-Free Methods

A polymer MN fabrication technique based on a drawing lithography process [36] has been proposed as potentially a free mold and thus a less expensive solution to the mold-based techniques. This technique is based on the elastic response of the polymer material in its glass state. Melted polymer is dispensed on a fixed plate and elongated by drawing pillars in the upper-moving plate, generating the characteristic three-dimensional shape of the microneedle (Figure 1b). This technique, despite being free of master fabrication processes or replica molding, is affected by the limits arising from the high process temperatures. Further, the reproducibility of the features is lower than the stamp-based processes. The drawing lithography technique has been revisited by the droplet-born air blowing (DAB) method (Figure 1c) [37]. In the latter, drops of polymer solution are deposited in an array configuration on two plates. The plates are then contacted with each other and moved at a controlled rate. When the final distance between the plates is reached, the elongated polymer is hardened by means of air blowing, and the final microneedles are obtained. The droplet-born air blowing method has recently been combined with a cyclic contact and drying process on pillars (CCDP process) (Figure 1d) [38] in order to obtain dissolvable microneedles patches, characterized by the possibility of the rapid separation of microneedles from their backing film. Despite the fact that the DAB technique is free from the limitations of a high process temperature and UV, the process is still affected by the drawbacks associated with the dynamic interaction with the contact plate. Further, the proposed solution seems to be unsuitable for a uniform drug distribution within the microneedles, together with the non-ideal radius of curvature of their tips.

The absence of multi-step sequences, based on the molding processes, is one of the peculiar features of a novel fabrication technique for biodegradable polymer microneedles proposed by Vecchione et al. (Figure 1e) [39,40]. The basic principle of the fabrication technique is the pyroelectric effect of a dielectric crystal (lithium tantalate, LiTaO_3_); under an appropriate thermal stimulus applied to the crystal, an electric field is generated, which drives the microneedle drawing process. During the shaping process, the liquid cone solidifies following the fast solvent evaporation, in this way fixing the microneedle’s desired shape. The technology is free from a high process temperature and UV radiation, with the biopolymers processed in solution form at temperatures between 20 and 40 °C. Recently, this process has been improved in terms of shape by removing the presence of a pedestal below the microcones and in terms of parallelization which now needs to be automated in order to provide an industrial scalable process [32].

Among mold-free technologies, additive manufacturing, or 3D printing, has been shown to be an emerging field in MN structure fabrication. In fact, employing Computer Aided Design (CAD) software allows the design of MNs with costumed density, length, and geometry, which can be printed with high reproducibility through the consecutive deposition of layers [42]. Additive manufacturing involves different technologies to produce MNs. Micro-stereolithographic 3D printing was used to create microneedle arrays, after having integrated the active agent into the polymeric matrix before photopolymerization [43,44]. Stereolithography has even recently been used to print pyramidal and conical shaped polymeric MNs for transdermal delivery. In this work, stereolithography was followed by inkjet printing to obtain drug-coated MNs [45]. 

Additive manufacturing is often combined with other technologies. For example, fused deposition modeling (FDM) was recently introduced to fabricate biodegradable polymer MNs (Figure 1f) and was combined with a chemical etching protocol to improve the feature size of the printed MN tips [41]. A more exciting approach relies on the use of a two-photon polymerization (TPP) 3D printing methodology to fabricate a refillable drug reservoir equipped with hollow MNs in microelectromechanical devices. This structure allows MNs to interface with larger delivery loads [46,47]. Equally interestingly, stereolithography 3D printing was employed to fabricate—in a single step—hollow MNs interfaced with microfluidic structures within a single device to obtain higher fluid management capabilities for transdermal drug delivery [48].

Additive manufacturing has been shown to be a promising and challenging technology to fabricate MNs and MN integrated devices. However, some limitations need to be overcome. First of all, there is a low availability of 3D printable biomaterials (with specific properties and viscosities at certain temperatures or photo-sensitivities) which are biocompatible and which have the appropriate biodegradation rate in the case of drug-containing materials. Additionally, the material needs to have good mechanical performance to allow MNs to pierce the skin. Lastly, the nature of the protein has to be considered: proteins can degrade when exposed to high temperatures or UV radiation.

## 3. Materials

**Figure 2:**
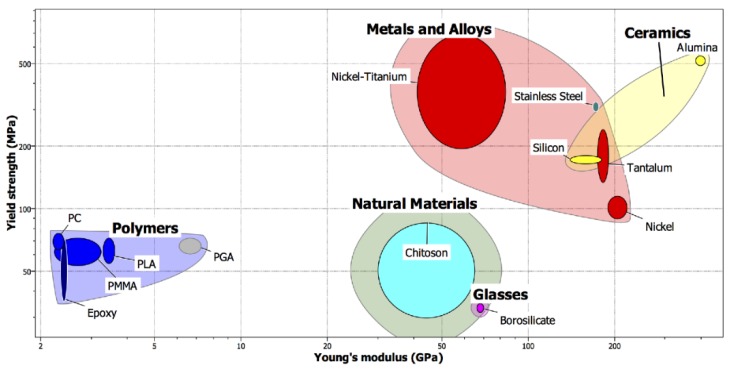
Yield strength vs. Young’s modulus of different materials used for the fabrication of microneedles. Reprinted with permission from [64].

Silicon, metal, silica glass, ceramics, and polymers have been employed to prepare MNs. The first study regarding silicon MNs to enhance drug delivery through the skin was published in 1998 [49]. The use of silicon has some limitations related to the intrinsic fragility of this material (some silicon MNs could fracture after insertion in the skin, causing the onset of silicon-based granulomas) and high production costs [50]. Some biocompatible metals have also been used. The most common metals are stainless steel, palladium, and titanium. They possess good mechanical properties, expressing Young’s moduli of 180, 117, and 110 GPa, respectively. These values are comparable with silicon for which the range is between 50 and 180 GPa. The first reported metal for manufacturing MNs was stainless steel [14]. Silica glass is another alternative, which is intrinsically physiologically inert. Since silica glass is brittle and not absorbable, like silicon, it can be used only for experimental purposes, not for commercial usage [51]. Some types of ceramics, such as alumina (Al_2_O_3_), calcium sulfate dehydrate, calcium phosphate dehydrate, and Ormocer can be used to manufacture MNs [52,53]. Carbohydrates such as maltose, sucrose, and trehalose can be used as a matrix of MNs [54,55]. They show high biocompatibility and safety for drug delivery applications [55]. Other carbohydrates, e.g., gum polysaccharides and hyaluronic acids (HA), with molecular weights higher than the previous saccharides, e.g., maltose, can be used in the matrix of MNs or as particles embedded in MNs [56,57,58,59]. Proteins, synthetic polymers, and polysaccharides exhibit high biocompatibility and degradability. The most frequently used matrix materials include poly-L-lactic acid (PLA) [60], polymethylmethacrylate (PMMA) [61], poly(lactic-co-glycolic) acid (PLGA) [62], poly(vinylpyrrolidone) (PVP) [29], and poly(vinylalcohol) (PVA) [30] (Figure 2). Synthetic polymers such as poly(methyl vinyl ether-*alt*-maleic anhydride) (Gantrez AN-139) or poly(methyl vinyl ether-*alt*-maleic acid) (Gantrez S-97^®^) have also been employed [63].

## 4. Penetration and Mechanical Characterization Tests of Microneedles

The human skin plays a pivotal role in protecting our bodies against pathogens and external toxic molecules [65]. The surface area of human skin in adults is between 1.5 and 2.0 m^2^, whereas skin thickness varies among the different body parts and between men and women and young and old individuals. For instance, the thickness of the forearm skin in males and females is 1.3 mm and 1.26 mm, respectively. Generally, the skin is made of three main layers: the epidermis, dermis, and hypodermis [66]. The outer layer (epidermis) acts as a barrier that protects humans from infections and adjusts the amount of water released from the body. The second layer (dermis) is located between the epidermis and the subcutaneous tissues and is divided into the papillary region and reticular dermis. The hypodermis is the deepest and thickest skin layer and it contains fibroblasts, fat cells, connective tissue, larger nerves or blood vessels, and macrophages. The hypodermis thickness differs in various areas of male and female bodies. 

Thus, according to human skin anatomy considerations, tests for studying the mechanical properties of MNs for insertion in human skin are unavoidable [67,68]. MN mechanical characterization is an inevitable step in the successful development of MNs. MNs are subjected to a variety of stresses owing to the human skin’s non-uniformity. Therefore, having a standard inherent strength is essential to prevent MN fracture due to bending, buckling, and baseplate fracturing [69]. Generally, there is no specific test to demonstrate the good mechanical performance of MNs for in vivo skin insertion. Consequently, MNs’ mechanical characterization involves a series of tests [70]. To evaluate the mechanical behavior of polymeric MNs with different geometries, increasing the probability of successful MN insertion, broad tests are desired [63], including axial and transverse loadings.

The *axial fracture forces test* is usually employed to measure the MN mechanical strength [67]. The maximum force applied exactly before dropping is considered to be the force of needle failure. Axial compression tests involve a force applied in parallel to the microneedle axis [65]. They usually require the employment of a mechanical test station that records both displacement and force while the MNs are pushed against a hard, metallic surface at a distinct MN row [19].

The *transverse failure force (TFF) and shear strength tests* are essential to provide a comprehensive profile of microneedle behavior during their applications, because skin surface irregularity often causes the imperfect penetration of MN arrays, causing MN transverse bending [70]. In this test, a mechanical station is usually employed where a transverse force is applied at a distinct MN row (5–10 MNs in a row) until the MN breaks. An unexpected fall in force corresponds to MN failure. TFF testing of a MN row in an array includes dividing the force needed for the failure of all MNs within the row by the MN number to estimate the transverse failure force per single MN [63,70]. A limitation of this test is the difficulty in the manual alignment of the probe with a distinct length on the MN because of the micron-scale of MNs [64]. 

On the other hand, the main challenge in using MNs is to transport biomolecules into the skin in an efficient and reproducible way. A lot of improved techniques in combination with MNs have been investigated to enhance drug delivery after MN insertion. For instance, Wu et al. [68] studied the in vitro delivery of high molecular weight fluorescein isothiocyanate (FITC)-dextran derivatives, combining the effect of MN pre-treatment and iontophoresis (ITP). Results showed that a significant intensification in FITC-dextran penetration was observed when MNs and ITP were combined. Unfortunately, this strategy is invasive and generates poor patient compliance [68]. Better results were obtained by a novel dissolving microneedle (DMN) called the “Troy microneedle”. Kim et al. [38] demonstrated that the traditional patch-based DMNs failed because of insufficient skin insertion and rapid separation of microneedles due to their strong bond with the supporting material. On the contrary, the Troy microneedle, created by cyclic contact and drying on the pillar (CCDP), is able to generate complete and rapid delivery of the encapsulated drugs. In particular, in vivo skin penetration studies demonstrate that the development of microneedles on pillars produces a wild separation without waiting for the dissolution of the polymer matrix. This feature allows the complete delivery of the drug into the skin, overcoming the viscoelastic skin barrier [38]. Although the Troy microneedle goes beyond the limits of the low penetration efficiency of the patch-based DMNs, many studies have yet to be implemented to achieve an optimal DMN-mediated therapy. Several other aspects must be considered in the field of microneedle penetration [71]. For example, penetration is mainly attained by using sharp-tipped needles with an appropriate length to overcome the bending of the skin’s compliant surface that takes place before penetration. Numerous factors affecting the MN insertion depth have been examined. The MN insertion depth increases with increased applied velocity and applied force. The MN length also influences the insertion depth, in contrast to MN interspacing. Needle reliability during penetration has been mainly attained by minimizing the required insertion force by using sharp-tipped needles and by maximizing the mechanical strength through increasing the Young’s modulus and needle diameter [62]. To determine whether polymer MNs are strong enough to insert into the skin without fracturing, it is necessary to determine the force needed to cause needle fracture by axial loading measures as a function of the needle length, base diameter, and Young’s modulus. The fracture force decreases with an increasing needle length. On the other hand, the fracture force increases with an increasing base diameter. The fracture force in polymer MNs increases with an increasing Young’s modulus, due to the employment of polymers with greater mechanical strength which have larger failure forces. 

## 5. Type of MNs Based on Their Structure and Release Profile

MNs can be classified into solid MNs and hollow, coated, dissolvable, degradable, and swellable systems. Recently, some responsive polymers were embedded into MNs in order to achieve an on-demand release [64]. Solid MNs are the first generation of MNs and are generally fabricated with silicon or metals [67,70]. According to the MN release profile, there are MNs that exist for either burst (instant) release or prolonged release. 

For example, prolonged release can be achieved by encapsulating the drug in polymeric microparticles/nanoparticles and hydrogel-forming structures, while burst release can be performed using dissolvable matrices. Furthermore, MNs, as intelligent carriers, are responsive to internal and external stimuli and have been designed to have a smart drug delivery system. 

### 5.1. Hollow MNs

Hollow MNs emerged to inject liquids [72] and suspensions for drug infusion into the body through the needle bore. Silicon, metal, or glass can be employed for the preparation of hollow MNs with an adjustable bore diameter. A study using ovalbumin (OVA)-loaded PLGA nanoparticles (NPs) delivered by hollow MNs showed a higher antibody response and a higher amount of interferon-γ as compared to intramascular NP injection and soluble antigen delivered by hollow MNs [73]. 

In a recent study, four types of nanoparticles (NPs) including PLGA, liposomes, mesoporous silica, and gelatin NPs delivered by hollow MNs were used to encapsulate OVA and its adjuvant in order to evaluate the different physiochemical properties of the NPs. PLGA NPs, particularly cationic liposomes, induced the highest immune responses, arising from the strong interaction between the antigen/adjuvant and the nanoparticle matrix. Gelatin and mesoporous silica have faster release, which may be due to the weak electrostatic interaction between the antigen/adjuvant on the surface of NPs. Antigen co-encapsulated with adjuvant induced antibody responses in a more effective way compared with the free OVA/adjuvant. This demonstrated that hollow MNs accompanied by the applicator are a promising tool for intradermal vaccination [74]. Using the same liposome composition and the co-encapsulation of diphtheria toxoid and adjuvant, it was demonstrated that cationic liposome is able to initiate strong immune responses. The intense interaction between a positive liposome and negatively-charged cell membrane allowed sustained release of the antigen and adjuvant [75]. Obviously, it is desirable for hollow microneedles to possess acceptable mechanical strength to avoid breakage into the dermis and to ensure that the bores are not blocked during transdermal drug delivery. Although a number of good fabrication techniques have been developed during recent years, hollow MNs still show some limitations. First of all, they are characterized by having possible allergic reactions in the case of metal MNs [13,14,76] and require specialized personnel and a complex pump-based set up for their injection [77]. Despite this, some studies recommend their applications in the dermatological field as well as in clinical applications for local and systemic delivery of drugs, vaccines, and cells [77,78,79].

### 5.2. Coated MNs

**Figure 3:**
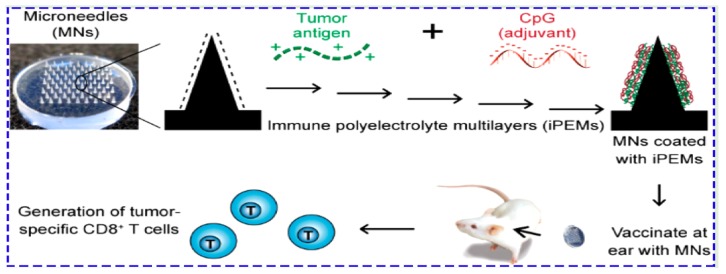
Assembly method for immune polyelectrolyte multilayers on microneedle arrays to enhance cancer vaccination. Reprinted with permission from [102]. Immune polyelectrolyte multilayers (iPEM), cytosine triphosphate deoxynucleotide guanine triphosphate deoxynucleotide (CpG).

In this kind of patch, drugs are adhered directly onto the surface of solid or polymeric MNs. Since the coating layer reduces the mechanical strength and sharpness of the needles, the drug loading on the needle surface is limited to a low amount. As a consequence, coated MNs are only usable for some specific applications in which a low dose is needed [80,81]. Human growth hormone ormone-coated titanium MNs showed a bioavailability similar to that of subcutaneous injections [82]. In another study, interferon-alpha coated polymeric MNs induced an antitumor effect in cancerous mice, similar to subcutaneous injections [83]. Parathyroid hormone delivery by utilizing coated MNs showed a rapid peak in plasma, faster than subcutaneous injections, with a high temperature stability for more than 2 years [84]. Immune polyelectrolyte multilayers adhered on MNs can be used for delivering human melanoma antigens and as a potent toll-like receptor adjuvant. In particular, the layer-by-layer deposition of vaccine components, consisting of tumor peptides and adjuvants, on MNs initiated the tumor-specific T cell response and led to a recall responses (Figure 3) [84]. The H1N1 influenza vaccine coated stainless steel MNs to trigger an immune response in young mice, indicating that MN patches are able to induce higher levels of functional antibodies as compared with a group immunized intramuscularly [85,86,87,88,89,90,91,92,93,94,95,96,97,98,99,100,101]. 

### 5.3. Dissolvable Matrix MNs

Dissolving MNs were created to encapsulate drugs within a water-soluble polymer matrix, and they become completely dissolved once the MNs are inserted into the skin, with a dissolving time from minutes to hours [103,104]. Sullivan et al. [105] designed dissolvable polymeric MNs using polyvinyl pyrrolidone (PVP) as a matrix to encapsulate the influenza virus. In order to evaluate antigen stability, mice were immunized intramuscularly with an inactivated influenza virus or via a microneedle patch encapsulating the same amount of virus. Results suggested that a single dose with dissolving MNs induces a superior immune response to those obtained with intramuscular injections [105].

Additionally, for influenza vaccination, materials such as trehalose [99] and carboxymethyl cellulose [106] have been used to create dissolvable MNs. Hyaluronic acid (HA)-based MNs have been exploited for tetanus, diphtheria, influenza, and malaria [107]. Ling et al. [108] designed a dissolvable microneedle for insulin delivery with a starch/gelatin matrix, which was able to promote rapid and efficient delivery into the skin for diabetes treatment.

In the direction of burst release, HA-MNs were loaded with an amyloid-β 42-amino acid peptide antigen to develop a vaccine for Alzheimer’s disease. The patients showed an efficient immune response after MN application [109]. The incorporation of some excipient into an inactivated polio vaccine is capable of improving the thermal stability as compared to the liquid inactivated polio vaccine [110]. The storage condition and thermal characterization of the MNs are discussed in Section 6. Dissolvable MNs are not the ideal system when prolonged protein release is needed [111].

### 5.4. Degradable Particle Embedded-MNs

**Figure 4:**
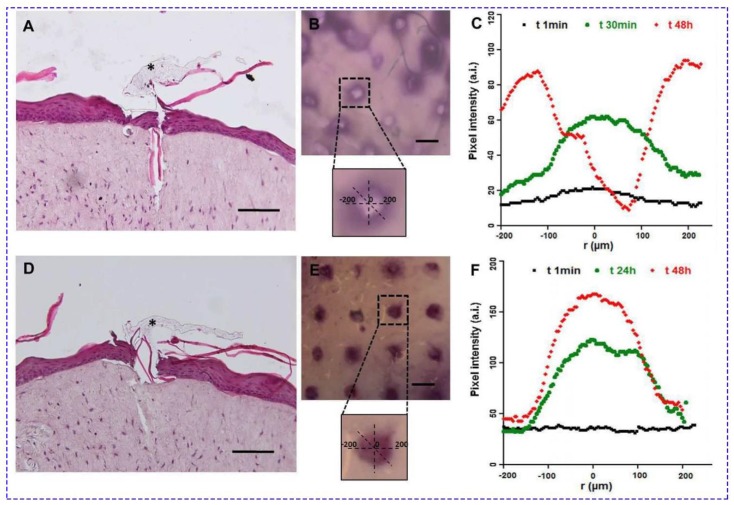
Functional test of MNs in a full-thickness human skin model. The pictures in (**A**–**C**) refer to the encapsulating enzyme in the tip; (**D**–**F**) refer to enzyme-encapsulated microparticles. A D Histological images after 48 h; black asterisks indicate the polyvinyl pyrrolidone (PVP) polymer remained after removing the patch (scale bar = 100 µm). B E Stereomicroscopic images of Endo-Human Skin Equivalent (Endo-HSE) (histological) 48 h after indentation (scale bar = 500 µm). The inserts of the stereomicroscopic images are the schematic representation of the methods used to calculate the diffusive radius reported in the *x*-axis of the successive graphs. (**C**,**F**): The graphs plot, at three time points, the pixel intensity as it corresponds to the concentration of the substrate oxidation product diffusing into the extracellular matrix vs. the radius of the diffusion pattern. Reprinted with permission from [62].

In order to achieve sustained drug release, MNs with biodegradable polymer particles were designed. The drug payload is gradually released by simple diffusion and hydrolysis of the polymer [62]. PLGA is the most common biocompatible and biodegradable polymer used to encapsulate drug/therapeutic agents [112]. Encapsulating labile molecules in microparticles (MPs) and NPs has great advantages, particularly if compared to soluble antigen formulations [113]. Embedding antigen in particles can retain the antigen activity from enzymatic degradation and increase the uptake by antigen present cells (APCs) in a targeted and sustainable manner, while restricting the entry of encapsulated antigens to the systemic circulation [114]. Additionally, particulate antigens are more efficiently cross-presented via MHCI molecules to CD8+ T cells than soluble antigens. This allows the simultaneous induction of both CD4+ as well as robust CD8+ T cell responses. In a study, OVA-loaded NPs were prepared by the (water in oil in water) W_1_/O/W_2_ emulsion method and successfully introduced into the microneedle patches [115]. The results showed the generation of potent CD8+ cytotoxic T cell and CD4+ Th1 immune responses against an encapsulated antigen. Degradable and dissolvable MNs, developed in order to tune the payload release, were reported by Battisti et al [62]. In this system (Figure 4), not only the microparticles, but also the hydrophilic tip, are able to entrap therapeutic agents, leading to a bi-compartmental system. In this method, firstly, a highly soluble polymer such as PVP or HA is spin-coated on the stamp, and then, microparticles are introduced in the cavities of the mold. 

**Figure 5:**
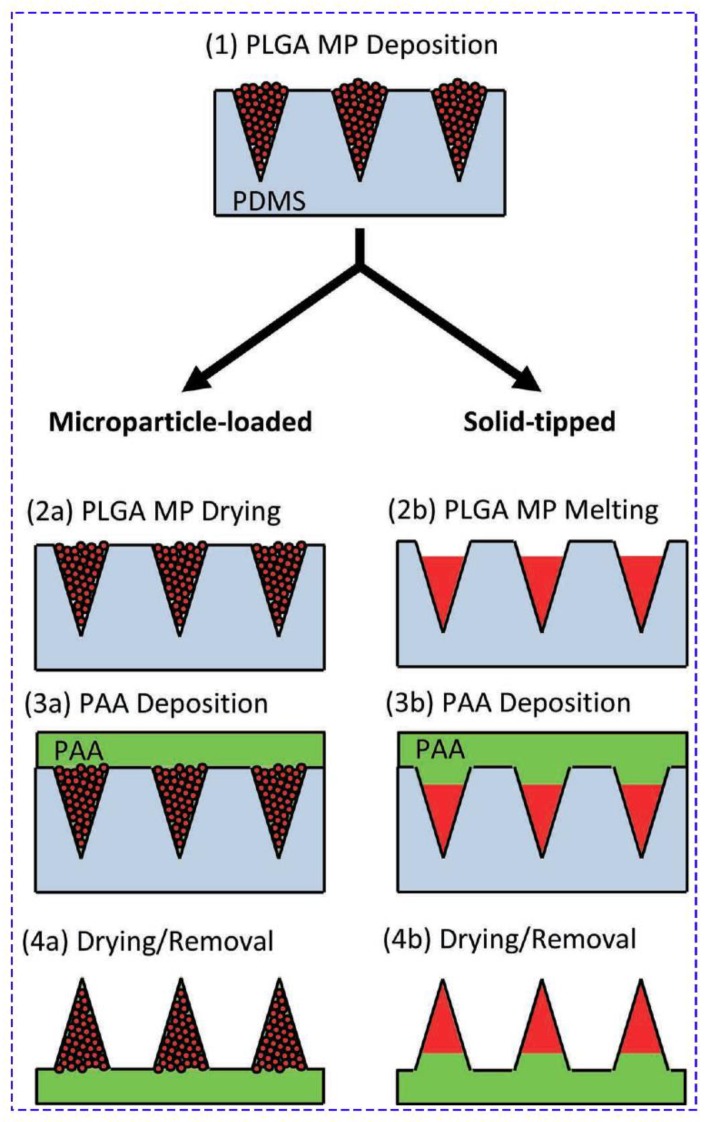
Schematic view of composite microparticle and bulk poly(lactic-co-glycolic) acid (PLGA) tip MN fabrication. Molds were first filled with PLGA microparticles (1). PLGA microparticles were then either dried in mold cavities (2a) or fused at a high temperature to create a solid tip (2b). Concentrated aq. poly(acrylic acid) solution was then centrifuged onto the filled molds to create a supportive matrix (3a) or pedestal (3b) for rapid dissolution in vivo. After drying, MNs were removed from molds (4a, 4b). Reprinted with permission from [117]. poly(lactic-co-glycolic) acid (PLGA), MP (microparticle), PDMS (polydimethylsiloxane), PAA poly(acrylic acid).

Much effort has been made to solve the deactivation of protein structures during the fabrication steps. In this context, ovalbumin (OVA), as an antigen model, was loaded in PLGA microparticles by the self-healing method. This method guarantees the stability of the antigen structure since microparticles are incubated with the antigen solution, preventing the antigen from exposure to the mechanical stresses present during the preparation process [116]. Long term delivery has been gained through the PLGA microparticle/poly(acrylic acid) composite MN arrays. Implanting the microparticles or solid polymer MNs in the tissue gives improved cellular immunity and equivalent generation of serum antibodies as compared with traditional needle-based vaccination (Figure 5) [117].

In the context of the prolonged release of the vaccine, MNs composed of a silk tip supported on a poly(acrylic acid) PAA base have been designed. PAA bases dissolve quickly to deliver the antigen while also implanting silk hydrogel depots for sustained cutaneous release over 1–2 weeks. Microneedle arrays containing OVA loaded in the silk needle tips or OVA in the PAA pedestals allow for bolus and sustained release [118]. The use of a complex system such as the degradable particle embedded-MNs is justified in the case of having a need for an engineered release profile.

### 5.5. Swellable MNs

**Figure 6:**
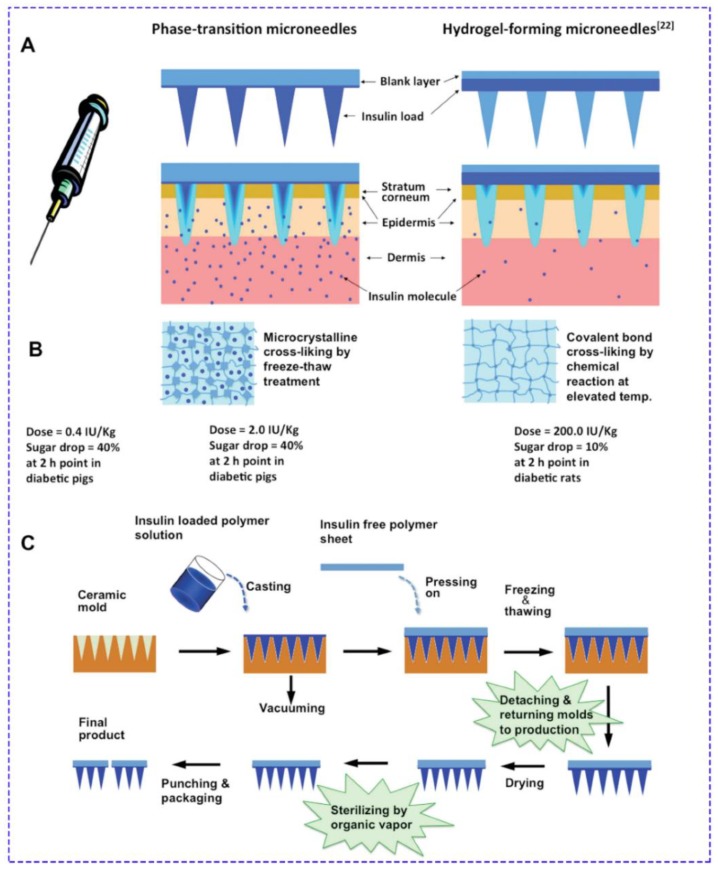
Fabrication process of phase transition patches. (**A**) The MNs absorb the interstitial fluid (IF) from the dermis layer to convert from a hard, glassy state to a hydrogel state to allow the preloaded insulin to release to the bodily fluid in the dermis layer. (**B**) The microneedle matrix of phase-transition microneedles (PTM) is cross-linked through microcrystalline domains as the cross-linking junctions via a freeze–thaw treatment to avoid dissolution, while that of hydrogel forming is cross-linked through covalent bands as the cross-linking junctions via a chemical reaction. Therefore, insulin can be loaded in the needle tips of PTM to achieve a relative bioavailability of 20%, while insulin has to be loaded at the back of the microneedle array of hydrogel-forming microneedles (HFMs), leading to a bioavailability of less than 1% due to the extended diffusion pathway. (**C**) The PTM patch may be fabricated using a scalable process comprising a sequence of simple unit operations involving the circulation of the molds in the production line and sterilization of the final product by steaming in oxirane vapor. Reprinted with permission from [123].

Swellable MNs, also known as cross-linked hydrogels, are solid systems that swell after the uptake of interstitial fluid (IF), after which they deliver the payload, and are removed intact from the skin [119,120]. These swellable MNs were introduced in 2012 and contain poly(methyl vinyl ether/maleic acid), cross-linked with poly (ethylene glycol) (PEG) to deliver bovine serum albumin (BSA) [121]. Polyvinyl alcohol (PVA) is also a well-known polymer that can generate a swellable system with a unique phase-transition property during temperature change [122]. Unlike chemical cross-linking, phase-transition MN patches (Figure 6) are able to generate microcrystalline domains as a junction by a freeze–thaw method [123]. The microcrystalline cross-linking is able to encapsulate insulin, free of hazardous cross-linking agents, which typically is needed for chemical and ionic cross-linking. The applications of swellable MNs are not just limited to drug delivery but can also be used to extract IF for subsequent analysis [124]. Designing bullet-shaped double-layered MN arrays with water-swellable tips allows adherence to the skin due to the formation of a desirable structure for interlocking with tissues [125]. Insulin-loaded swellable MNs exhibited sustained release, allowing a gradual decrease in blood glucose levels [125]. Delivery of the anticancer drug bevacizumab with dissolving and hydrogel-forming MNs has been investigated. The results demonstrate a better performance of the hydrogel-forming MNs as compared with the dissolving MNs. The maximum concentration of bevacizumab in the serum was gained 1 day after having removed the hydrogel from the skin, suggesting that the controlled delivery via this system is attributed to the ability of bevacizumab to enter the microcirculation from the skin [126].

Some limitations regarding swellable MNs are related to the need to keep the patch on the skin for all release timeframes. Additionally, particular attention has to be given to the choice of cross-linkage reaction, which needs to be biocompatible. 

### 5.6. Bio-responsive MNs

Bio-responsive materials have the outstanding characteristic of sensing physiological or pathological signals and regulating on-demand release based on the presence and intensity of a specific stimulus [127]. One of the most important proteins that needs to be released with respect to a stimulus is insulin. In fact, normal insulin secretion from β-cells in the pancreas regulates the release based on blood glucose levels (BGLs) [128]. Obviously, the exogenous source should be able to release the therapeutic in high BGLs, imitating physiological dynamical insulin secretion to avoid dangerous situations like hyper/hypoglycemia [129]. Combining glucose-responsive materials with MNs creates a smart and effective insulin delivery method. In this regard, various glucose-responsive materials have been incorporated into the MN matrix [130] or in particles embedded in MNs [131]. For instance, non-degradable MNs were constructed from hydrogel containing phenylboronic acid (PBA) as a glucose-sensitive agent. The results exhibited not only that insulin release depended on BGLs, but they also showed the stability and shape preservation after 7 days in aqueous solution, which demonstrated their capability for long-term, on-demand insulin release [132].

**Figure 7:**
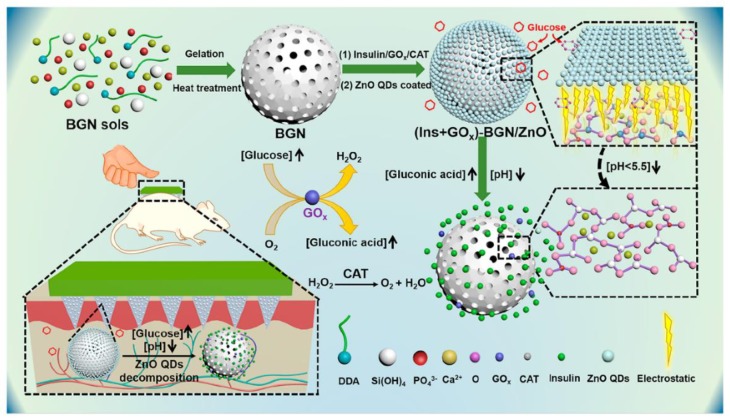
Bioactive glass nanoparticles (BGN) can be manufactured by BGN soles through gelation under high temperatures. These BGNs were filled with GOx/catalase (CAT) inside their pores, and then the NPs were coated by ZnO quantum dots (QDs). As BGLs grow, the pH drops to a value lower than 5.5 as a result of the reaction occurring by GOx. ZnO QDs are dissolved under this low pH, which disintegrates the BGN, and thus, insulin is free to be released from the disassembled particle. The catalase (CAT) enzyme is responsible for reducing the harm caused by H_2_O_2_ on the surrounding tissue. Reprinted with permission from [134].

Applying another strategy, Juiang et. al. created polymeric MNs loaded with insulin containing glucose-responsive mesoporous bioactive glass nanoparticles (BGNs). The silica NPs were coated by a pH-responsive material and glucose oxidase (GOx), which made them link BGLs as the pH changed, consequently giving the ability of glucose level sensing. When the pH drops because of the high concentration of glucose, this system can sense pH alteration through the pH-responsive material and lead to insulin release [133]. In another study, insulin loaded mesoporous bioactive glasses (MBGs) containing GOx were capped by ZnO quantum dots (ZnO QDs). Since ZnO QDs are dissolvable at a low pH, they can act as pH-sensitive agents (Figure 7). In vivo studies showed the achievement of prolonged moderate blood glucose control by the glucose-responsive MNs, while BGLs dropped quickly when subcutaneous injections were used [134].

**Figure 8:**
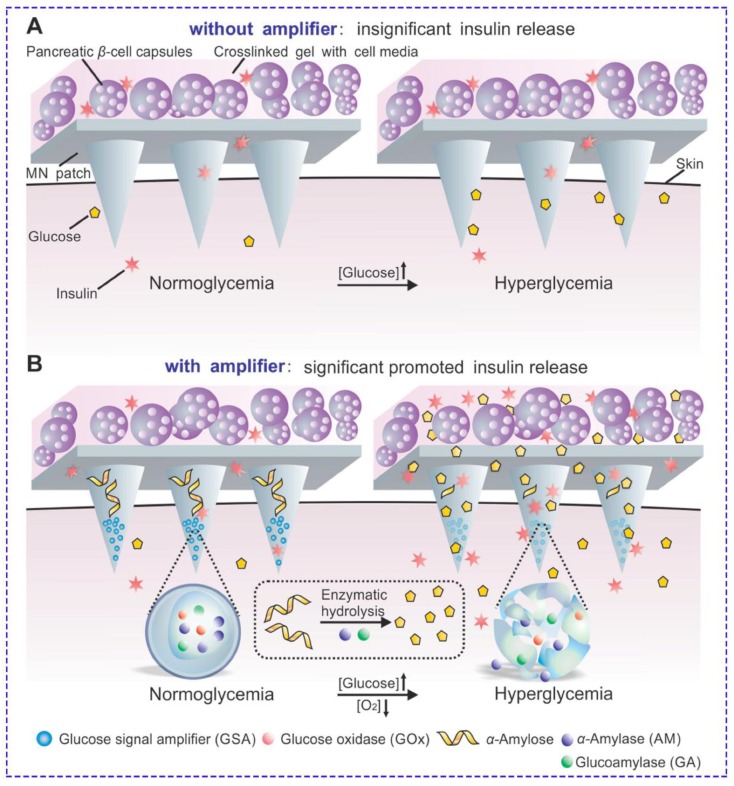
(**A**) Live glucose-responsive design based on the diffusion of glucose in cross-linked hyaluronic acid and stimulation of cells. Unfortunately, no significant release was observed at different glucose concentrations due to the low glucose diffusion in MNs. (**B**) Living-synthetic responsive system with amplified glucose levels, aided by nanovesicles containing three enzymes. After sensing local hyperglycemia, the reaction by glucose oxidase occurs, which results in a decrease in pH and leads to low O_2_ levels. Thus, hypoxia-sensitive nanoparticles dissociate, resulting in the release of all enzymes. Released α-amylase and glucoamylase convert α-amylose to glucose in two separate steps. Higher amounts of glucose diffuse and make the living part of the system secrete insulin effectively. Reprinted with permission from [135].

Glucose control can also be achieved by live (cell-based) and synthetic glucose-responsive systems, for instance, glucose-responsive MNs based on cross-linked hyaluronic acid with β-cells encapsulated in microgels on the tip of MNs (Figure 8). Amplified glucose levels can effectively diffuse and activate the release of β-cells on the tips of MNs. The amplified signal showed significant effective glucose control for almost 8 h compared with other treatments lacking the live/synthetic part or even with one of the enzymes involved [135].

Bio-responsive MNs are the state-of-the-art MNs, but again, their complexity needs to be justified. Additionally, they need to be kept on the skin for the release timeframe, but this is not a limitation in applications, such as insulin delivery, where the use of a reservoir is needed anyway to guarantee the amount of the molecule being delivered. 

## 6. Characterization of MNs and Storage

Mechanical strength and indentation ability are strongly dependent on the amount of water residual in the polymeric MNs. For this reason, here, we take a brief look at the characterization of MNs. For instance, the addition of sucrose to the dissolvable MNs can change the mechanical properties. Also, the addition of sucrose results in the faster release of sucrose/dissolvable MN formulations compared with MN formulations without sucrose [136]. Unlike low-molecular-weight drugs, proteins are sensitive to pH, temperature, or fabrication procedures, needing mild manufacture approaches. 

### 6.1. Biomolecule Activity 

An encapsulated biomolecule does not have the same stability and sensitivity to temperature, humidity, and shelf life in solvent as a free biomolecule. For example, proteins, antibodies, and viruses are very susceptible to degradation in comparison to organic molecules; in particular, proteins are vulnerable if they are exposed to some organic solvents, such as dichloromethane, ethyl acetate, and dimethylcarbonate (DMC), which are used during PLGA microparticle production by the double emulsion technique. Since the liquid and commercially-available lyophilized drugs lose their activity after preservation at room temperature, the thermostability of MNs is great of interest. The common fabrication methods employed for protein-loaded MNs involve the use of high temperatures, a vacuum, or exposure to an ultraviolet source, which may be harmful to protein activity [137]. Another approach is based on the encapsulation of the drugs into a polymeric matrix, offering the advantage of higher drug loading in a single step [138]. Indeed, the encapsulation of proteins inside micro/nanomaterials has been already studied using several methods, such as single/double emulsion, microfabrication, and electrospray [139,140,141]. For instance, lipid nanocarriers can be used to encapsulate proteins for homogenizing a mixture of lipids hydrated with protein solutions [142]. In this regard, therapeutic proteins such as insulin or inactivated influenza vaccines can be encapsulated in microneedles. In particular, microneedles fabricated using a biocompatible polymer were able to encapsulate the inactivated influenza virus vaccine, generating, after skin insertion, a robust antibody immune response in mice and providing complete protection against influenza [105]. In another study, insulin-loaded microneedles were prepared using dextrin to ensure the percutaneous administration of insulin; this formulation was able to maintain the active insulin after 1 month of storage at different temperatures ranging from −80 to 40 °C [143]. Other studies found that using dissolvable MNs made of a starch/gelatin matrix enhances the stability of insulin at room temperature or slightly higher for at least 1 month [108]. 

Recently, Yang et al. [9] developed a photolithography-based method, utilizing low exposure to ultraviolet light for the encapsulation of BSA in MNs. They tested the stability of the protein by analyzing its primary, secondary, and tertiary structures, reporting complete stability of the protein under working conditions. In addition, embedding Human Growth Hormone (HGH) in dissolvable MNs allowed for the maintenance of its complete activity after encapsulation and the retention of most HGH activity after storage for up to 15 months at room temperature and humidity conditions [103]. Other good results were obtained using hyaluronan-based microneedles loaded with immunoglobulin G (IgG). These MNs were able to efficiently penetrate and rapidly dissolve in the skin, preserving protein stability during the preparation process. In particular, protein stability and aggregation were investigated at the molecular, submicron, and micron-size levels [30]. Aside from this, some other studies addressed protein stability in microneedle formulations, emphasizing how this technology is suitable for proteins [62,144,145]. 

### 6.2. Sterilization and Storage

Sterilization, packaging, and storage should be taken into account for scaling up MNs before being commercialized. In contrast to conventional transdermal delivery platforms, MNs are not applied to the skin surface; indeed, MNs pierce the outermost protective skin layer, penetrating into the epidermis and dermis, which are normally sterile parts of the body [146]. Therefore, it is obligatory that MNs must not contain any microbial agents that can cause skin or systemic infections. In addition, the bioburden should be controlled to avoid stimulation of the immune cell population present in the epidermis and dermis [14]. Really, as stated by some published studies, the ability of microorganisms to cross the holes created in the skin by MN insertion seems to be negligible [147]. Some studies have demonstrated that by using solid silicon MNs, the crossing of *Candida albicans* and *Pseudomonas aeruginosa* pathogens through cell membranes is lower in comparison with the use of a 21G hypodermic needle [148]. Together with these results, another study performed by Wei-Ze et al. [149] reported that rats cured with solid silicon MNs did not become septic by *Staphylococcus aureus*. 

However, the best solution is to always subject the MNs to a complete sterilization process. One of the possibilities can be to use gamma irradiation after the complete fabrication of MNs. Several studies about hydrogel MN arrays showed that no measurable bioburden was detected in any gamma-sterilized devices, and endotoxin levels were below the Food and Drug Administration (FDA) limits (20 endotoxin units/device). Moreover, some types of MNs, e.g., hydrogel-forming MNs, are not affected by gamma irradiation (25 KiloGray (kGy)) in terms of their physical properties and drug bioactivity [146]. Another approach could be the use of materials that show antimicrobial properties, e.g., chitosan and its derivatives, as well as quaternary ammonium functionalized polymers [150,151,152,153]. In this case, additional safety concerns regarding the health of the selected materials must be addressed. The chosen materials should be biocompatible and safe in order to not develop local or systemic reactions, and there should be a balance between the antimicrobial activity and cytotoxicity [70].

However, the sterilization methods should be carefully chosen to avoid changing the product and increasing the manufacturing costs. For instance, aseptic manufacturing could be expensive, and heat or microwave heating could damage the MNs or their cargo. Alternatively, GMP productions would be needed to skip sterilization. Regarding storage, since MNs are highly sensitive to temperature and mechanical stress, it is necessary to keep the patches in a humidity-free state, e.g., in a desiccator. However, stability studies of long-term patches are needed. In this regard, Hiraishi et al. [154] reported that environmental humidity has an impact on the mechanical strength of the microneedle patches. The mechanical failure force test indicated that by increasing the level of humidity, the needle strength decreased. Wet conditions are also not suitable for maintaining the stability of proteins; indeed, the presence of an uncontrolled atmosphere can lead to protein unfolding, aggregation, or chemical degradation. Regardless, as previously mentioned, MNs do not need a cold-chain; storing at low temperatures is becoming a very useful platform for transporting drugs to undeveloped countries without the need to have trained staff, e.g., nurses and physicians [10].

## 7. Conclusions

The clinical development of proteins is faster than that of small-molecules or peptides [155], although their chemical-physical properties put wide restrictions on transdermal delivery [156]. Particularly, proteins are subject to degradation in aqueous environments, undergoing aggregation, denaturation, or precipitation mechanisms [157,158]. Microneedles possess many advantages to avoid protein denaturation and aggregation (e.g., rapid delivery) as compared with other systemic administrations, and for this reason, they could significantly revolutionize the field of drug delivery.

Unfortunately, they also have limitations, for example, MNs are still missing many requirements (e.g., safety, high therapeutic effect, drug effectiveness) to be defined as “smart devices” for biomedical applications. Moreover, great consideration should be given to reduce the skin allergies, redness, and irritation associated with MN treatments. Despite these restrictions, the growth in MN fabrication and biomolecule encapsulation processes make them a favorable platform for the dermal delivery of drugs.

## 8. Future Perspectives

Microneedles can be considered emerging devices in the field of drug delivery compared with current techniques. In fact, MNs have achieved excellent results, especially in the cosmetic field, undergoing rapid growth in the last decade. For example, HA-MN patches are currently sold around the world for cosmetic purposes by the Raphas company [159]. Moreover, multi-compartmental and novel smart materials, such as hydrogel MNs, can provide a designed drug release which, in the case of bioresponsive MNs, is even more advanced and effective [160]. Starting from these new proposals and developing progressively innovative technologies, microneedles could represent a significant improvement in the fields of drug administration and delivery, disease treatment, and cosmetic applications. Of course, on an engineering level, the complexity of the system needs to be justified by the final application, always respecting the market price expectations. 

## Abbreviations

APC	antigen presenting cells
aPD1	anti-programmed death-1
BGLs	blood glucose levels
BGs	bioactive glass nanoparticles
BSA	bovine serum albumin
DDCs	dermal dendritic cells
DC	dendritic cell
DMNs	dissolvable microneedles
HA	hyaluronic acid
IF	interstitial fluid
iPEMs	immune polyelectrolyte multilayers
MBGs	mesoporous bioactive glasses
MNs	microneedles
MPs	microparticles
NPs	nanoparticles
OVA	ovalbumin
PBA	phenylboronic acid
PEG	poly (ethylene glycol)
PVA	polyvinyl acetate
PVP	polyvinylpyrrolidone
RF	radiofrequency
TDD	transdermal delivery
TFF	transverse failure force
TGA	thermal gravimetry analysis
W/O/W	water-in-oil-in-water emulsion method
